# Obituary

**Published:** 1893-04

**Authors:** 


### In Memory.

### Obituary.

The estimable wife of Dr. Charles R. Butler died at her home in Cleveland, O., March 1st, 1893. She was a noble character. Her eulogy was her life in deeds of charity and steadfast loyalty to the church of her choice. Her father, Ira Eddy, was an active itinerant minister of the M. E. Church for more than fifty years. The sincere sympathy to Dr. Butler of his many friends may be expressed as did the Cleveland Dental Society in the following resolutions:

*Whereas*, It has pleased an allwise Providence to visit upon one of our most respected members, Dr. C. R. Butler, a sad affliction by removing his beloved wife, therefore be it

*Resolved*, That in this hour of bereavement we deeply sympathize with himself and relatives in the great and irreparable loss which they are called upon to sustain.

*Resolved*, That these resolutions be spread upon the minutes and a copy be forwarded to Dr. Butler by the secretary.

H. L. Ambler,
S. B. Dewey,
W. H. Whitslar,
Committee.

